# The Effects of Light-to-Moderate Alcohol Consumption on the Cognitive Function of Community Nondemented Male Elderly: A Cohort Study

**DOI:** 10.1155/2021/5681913

**Published:** 2021-03-25

**Authors:** Zhang Yan, Zhang Yingjie, An Na, Qiu Qi, Li Wei, Wang Wenzheng, Sun Lin, Xiao Shifu

**Affiliations:** ^1^Department of Geriatric Psychiatry, The Third People's Hospital of Fuyang, Hangzhou, China; ^2^Department of Addiction Medicine, An Ding Hospital, Capital Medical University, Beijing, China; ^3^Alzheimer's Disease and Related Disorders Center, Department of Geriatric Psychiatry, Shanghai Mental Health Center, Shanghai Jiao Tong University School of Medicine, Shanghai, China

## Abstract

**Aim:**

To investigate the effects of light-to-moderate drinking on the cognitive function of the elderly in a large elderly community cohort. Although heavy drinking is linked with impaired brain functions, the effects of light-to-moderate drinking on the cognitive function of the elderly are still controversial.

**Methods:**

A total of 1469 nondemented elderly men from 15 research centers in 8 cities and provinces were included and divided into two groups: drinking (531 subjects) and nondrinking (938 subjects). Cognitive functions were assessed by the Beijing version of the Montreal Cognitive Assessment (MoCA) at baseline and one-year follow-up.

**Results:**

There was no difference in total cognitive scores between the light-to-moderate drinking and nondrinking groups at baseline and follow-up. Nonalcohol users performed better naming and abstraction function at baseline and better naming function at follow-up. There was no difference in cognitive performance decline and new-onset dementia rates at follow-up.

**Conclusions:**

Light-to-moderate alcohol consumption had no significant impact on the overall cognitive function and the risk of dementia in elderly men.

## 1. Introduction

Drinking is a common behavior worldwide. The World Health Organization (WHO) released a global status report on alcohol and health, which showed that about 3 million deaths were attributed to drinking in 2016; among those, 28.7% were due to injuries, 21.3% due to digestive system diseases, 19% due to cardiovascular diseases, 12.9% due to infectious diseases, and 12.6% due to cancer. Additionally, 49% of the Disability-Adjusted Life Years (DALYs) are caused by drinking [1].

Acute effects of alcohol could impair cognitive abilities, such as attention, psychomotor speed, tracking ability, working memory, and cognitive flexibility. Changes in cytokine levels induced by alcohol abuse affect inflammatory pathways in specific brain regions, such as the hippocampus and prefrontal cortex, which induce cognitive impairment in susceptible individuals [2]. Some studies showed that female heavy drinkers had severe cognitive deficits. For example, Scaife and Duka reported that binge drinking in adult women was more associated with impaired cognitive performance than nonbinge drinking [3]. Townshend and Duka also reported that women with heavy drinking habits had impairment in memory and attention [4]. Previous studies observed a U-shaped relationship between regular alcohol consumption and cognitive function and stated that frequent heavy alcohol drinking decreased cognitive performance, while light-to-moderate drinking had protective impacts [5]. Neafsey and Collins reviewed 143 studies on the relationship between drinking and cognitive function and found that light-to-moderate drinking did not impair cognitive function in younger subjects and reduced the risk of dementia in older subjects [6]. Also, a British study on alcohol consumption and cognitive performance showed that moderate drinking was beneficial to the cognitive function of female drinkers, who performed better in various cognitive areas [7].

Heavy alcohol consumption was considered harmful for brain function; however, the effects of light-to-moderate drinking in the elderly are still controversial. The current study was aimed at estimating the difference in cognitive function between light-to-moderate drinking and nondrinking and explore the risk factors among demographics, physical condition, and drinking status for the cognitive function of nondemented male elderly through a large sample of community cohort.

## 2. Subjects and Methods

### 2.1. Subjects

Based on the screening of general information, medical history, physical and neurological examination, and face-to-face cognitive assessment, 1469 nondemented elderly males from 15 research centers in 8 cities and provinces were included and divided into two groups: a 531 light-to-moderate alcohol user group and a 938 nonalcohol user group [8]. The Beijing version of the Montreal Cognitive Assessment (MoCA) [9, 10] was used to measure cognitive function. The MoCA test consisted of 30 items measuring multiple cognitive domains including visual space, attention, calculation, abstract, language function, and memory.

All included subjects met the following criteria: (1) Han ethnicity, male, ≥60 years old; (2) no history of dementia; (3) no severe physical illness including nervous system disorders and acute or life-threatening diseases; (4) ability to cooperate to complete the study; and (5) denial of heavy drinking (<5 units/day). Exclusion criteria were a history of mental illness or other conditions that affect cognitive function. Absence of dementia was diagnosed according to clinical dementia rating (CDR) and cognitive scores. When CDR = 0, subjects were included in absence of dementia.

Based on self-provided drinking information, we divided subjects into two groups. One standard drink unit was defined as 14 g of pure ethanol, and consumption of <5 units/day was considered light-to-moderate drinking [11]. Nonalcohol users were defined as individuals who never drank or rarely drank (such as holiday drinking, lower one standard unit per time). The drinking group was required to provide the exact amount and types of alcohol consumed (Chinese baijiu, 40% of ethanol; beer, 3% of ethanol; wine, 11% of ethanol; yellow rice wine, 12% of ethanol). Total daily alcohol intake was then calculated. All subjects had signed informed consent before initiating the study, and ethical approval had been obtained from the Ethics Committee of the Shanghai Mental Health Center.

### 2.2. Methods

Face-to-face interviews were conducted to gather demographics, lifestyle, and physical illness information; then, neurological examinations and cognitive assessments were performed. Professional clinicians then reviewed all information for diagnosis. The current study did not include female elderly due to the limited sample size. Based on the amount of alcohol consumption, light-to-moderate alcohol consumers were included and evaluated at baseline and one-year follow-up.

### 2.3. Statistical Treatment

Demographic, lifestyle, physical disease, and cognitive scores were analyzed using variance analysis for continuous variables and a *χ*^2^ test for the categorical variables between the alcohol and nonalcohol groups. Differences in demographics, lifestyle, and physical illness were controlled as covariables. A general linear model was used to analyze the differences in cognitive function between the two groups after adjusting the covariables.

The correlation of alcohol consumption with cognitive scores was examined by Pearson's correlation and linear regression analysis. Logistic regression analysis was employed to analyze the risk factors for cognitive function. All statistical analyses used SPSS version 17.0 software with a two-tailed *p* value of 0.05.

## 3. Results

### 3.1. Analysis of the Cognitive Function between the Light-to-Moderate Drinking and Nondrinking Groups at Baseline (Tables 1 and 2)

At baseline, the drinking group had 531 subjects, and the nondrinking group had 938 subjects. Light-to-moderate drinkers accounted for 36.15% of all the participating elderly male subjects; 60.64% were Chinese baijiu drinkers, 8.66% drank wine, 12.62% drank beer, and 17.08% drank yellow rice wine.

After analyzing the demographic data (age and education), lifestyle (smoking and tea drinking), and physical illness (hypertension, hyperlipidemia, diabetes, and traumatic brain injury), we found out some differences between the two groups, including education (*F* = −5.87, *p* ≤ 0.001), smoking (*F* = 132.76, *p* ≤ 0.001), and tea drinking (*F* = 41.56, *p* ≤ 0.001). These factors were then considered covariables in comparing the MoCA scores. The results showed no statistically significant differences in total cognitive scores (baseline MoCA: *F* = 3.21, *p* = 0.073). Simultaneously, we analyzed eight MoCA subtests, with the above confounding factors controlled as covariables. The results showed a significant difference in the naming function (*F* = 5.82, *p* = 0.016) and abstraction (*F* = 4.70, *p* = 0.03) with higher scores in the nondrinking group (Table 1).

The correlation analysis between the amount of alcohol consumption and the total cognitive scores showed no correlation among all subjects. Additionally, the analysis of the relationship between the amount of alcohol consumption and eight subtests of MoCA showed that both language fluency (*r* = −0.102, *p* = 0.039) and abstraction (*r* = −0.105, *p* = 0.034) negatively correlated with the amount of alcohol consumed.

A linear regression model was used to analyze the risk factors of cognitive function with MoCA scores as the dependent variables and age, education, tea drinking, smoking, alcohol drinking, hypertension, diabetes, hyperlipidemia, and traumatic brain injury as covariables. The results showed that age, education, tea drinking, and hypertension all affected MoCA score, but not drinking alcohol. On the other hand, with the MoCA naming function and abstraction as dependent variables and the above factors as covariables, the results showed that drinking alcohol was a significant risk factor for MoCA naming function (*B* = 0.10, *p* = 0.016) and MoCA abstraction (*B* = 0.10, *p* = 0.031) (Table 2).

### 3.2. Analysis of Cognitive Function between the Light-to-Moderate Drinking and Nondrinking Groups at Follow-Up (Tables 2 and 3)

One year later, at the follow-up, 354 subjects in the drinking group and 666 subjects in the nondrinking group received cognitive assessments. There were 63.28% of Chinese baijiu drinkers, 9.04% of wine drinkers, 11.86% of beer drinkers, and 15.82% of yellow rice wine drinkers.

We analyzed the demographic (age and education), lifestyle (smoking and tea drinking), and physical illness (hypertension, hyperlipidemia, diabetes, and traumatic brain injury) of the two groups. We found differences in age (*F* = −2.20, *p* = 0.028), education (*F* = −4.44, *p* ≤ 0.001), smoking (*F* = 80.98, *p* ≤ 0.001), tea drinking (*F* = 32, *p* ≤ 0.001), and diabetes (*F* = 4.28, *p* = 0.039). With the above confounding factors as covariables, the results showed no significant difference in total cognitive scores at follow-up between the two groups (follow-up MoCA: *F* = 0.65, *p* = 0.421). Additionally, with the above different factors controlled as covariables, analysis of 8 MoCA subtests showed a significant difference in the naming function (*F* = 5.57, *p* = 0.018), with higher scores in the nondrinking group (Table 3).

There was no correlation between the amount of alcohol consumption and cognitive function at follow-up. Correlation analysis between the amount of alcohol consumption and eight MoCA subtests showed that abstraction (*r* = −0.143, *p* = 0.013) and attention (*r* = −0147, *p* = 0.011) were negatively associated with the amount of alcohol consumed.

In the end, a linear regression model was established, with the follow-up cognitive scores as the dependent variable, while age, education, smoking, tea drinking, hypertension, diabetes, hyperlipidemia, and traumatic brain injury as covariables. The results showed that drinking had no significant effects on total cognitive scores. After including the follow-up MoCA naming, abstraction, and attention scores as dependent variables separately, the results showed that age and education had significant influences on follow-up MoCA naming function, which demonstrated that drinking alcohol was a significant risk factor for follow-up MoCA naming function (*B* = 0.16, *p* = 0.003) (Table 2).

The score declines (follow-up score minus baseline score) in MoCA total, MoCA naming, and MoCA abstraction were all calculated in the same manner. The results showed no significant differences between the two groups (Table 3).

At one-year follow-up, through review and diagnosis by specialists, we found the new-onset dementia rates were 3.1% in the drinking group and 4.7% in the nondrinking group, which proved no significant difference (*F* = 1.40, *p* = 0.236) (Table 3). When setting the onset of dementia as the dependent variable and the above confounding factors as covariables, the logistic regression analysis showed that drinking had no significant influence on the onset of dementia (*B* = 0.441, *p* = 0.268).

## 4. Discussion

Three conclusions were made from this study: (1) at baseline and follow-up, there was no difference in the overall cognitive function between the drinking and nondrinking groups; (2) drinking influenced the naming function at baseline and follow-up, and nonalcohol drinkers performed better in the naming function; (3) results at one-year follow-up showed that light-to-moderate alcohol consumption had no significant impacts on the risk of developing dementia.

At present, only a few studies existed on the impacts of light-to-moderate alcohol consumption on the cognitive function in the elderly community in China. Some foreign studies indicated that light-to-moderate alcohol consumption was beneficial to cognitive function. Additionally, Solfrizzi et al. discovered that light-to-moderate alcohol consumption lowered the progression rate of dementia among patients with mild cognitive impairment (MCI) [12]. The Lothian Birth Cohort study indicated that moderate drinking improved cognitive ability in late adulthood, possibly due to cerebrovascular health improvement. Women's overall alcohol intake almost derived from wine, and in men, the effects differed by various types of alcohol. Wine and sherry were associated with better verbal ability, while beer was associated with worse verbal ability [13]. In the present study, only male subjects were included, and the types of alcohol drinking were mainly Chinese baijiu (60.64%), beer (12.62%), and yellow rice wine (18.07%), which contained a relatively high concentration of ethanol (>11%). The effect of wine drinking was minimal in this study.

On a seven-year follow-up study on the elderly without dementia, Ganguli et al. suggested that light-to-moderate drinking, compared to nondrinking, was associated with less cognitive decline [14]. Additionally, Rehm et al. found out that light-to-moderate alcohol intake during middle-to-late adulthood lowered the risk of cognitive impairment and dementia [15]. However, our previous research on the Shanghai elderly community indicated that light-to-moderate alcohol consumption did not affect cognitive function [16].

Head Magnetic Resonance Imaging (MRI) showed that the cortical thickness of the left temporal lobe was lower in alcohol drinkers [17], indicating that drinking had certain effects on the temporal cortex functions, such as auditory processing and language recognition. The present study provided evidence that the naming function was negatively affected by alcohol consumption, which was consistent with the results derived from MRI data. In other studies, however, there was some controversy over the effects of alcohol consumption on brain structure. Verbaten concluded that even light-to-moderate alcohol consumption caused brain shrinkage, white matter volume increase, and grey matter volume decrease [18]. Moreover, a longitudinal cohort study of 550 participants over 30 years of follow-up suggested that alcohol consumption dose-dependently increased the odds of hippocampal atrophy, and moderate drinkers had 3 times the chances of right hippocampal atrophy [19]. Paul et al. also found that alcohol consumption was not protective on brain volume; the more alcohol consumed, the smaller the brain volume was [20]. Another study, involving 609 adult alcohol users, found that a higher Alcohol Use Disorders Identification Test (AUDIT-C) score was linearly associated with a thinner cortex in certain brain areas [21]. On the other hand, Shokri-Kojori et al. compared the grey and white matter volumes between alcohol and nonusers over 65 years and found no significant difference [22]. Our previous study found that in 141 nondemented aging participants, the left superior-temporal gyrus was an age-sensitive region, and alcohol consumption was significantly associated with a thinner left superior-temporal cluster cortex [16]. Notably, the superior temporal gyrus was one of the gyri in the temporal lobe responsible for comprehension of language, which was consistent with the results in the current study.

The difference between the two groups in the present study reached statistical significance on MoCA naming function. However, some foreign studies stated that alcohol consumption improved cognitive function, which conflicted with our findings. This conflict was perhaps related to wine consumption, which is associated with better language function. Additionally, our study did not include elderly women who usually consumed wine far more than Chinese baijiu. Also, compared to other studies, different cognitive assessment tools were used in our study, which resulted in different sensitivity on cognitive function. Although the one-year follow-up was a relatively short time for assessment, we would continue to follow up the cognitive function of light-to-moderate alcohol drinkers for further study.

This study concluded that light-to-moderate alcohol consumption, at both baseline and follow-up, had no significant impacts on the overall cognitive function and dementia risk. However, drinking alcohol negatively affected MoCA naming function.

## Figures and Tables

**Table 1:** Demographic, lifestyle, physical illness, and cognitive function of community male elderly at baseline.

	Drinking (*n* = 531)	Nondrinking (*n* = 938)	*F* or *χ*^2^	*p* value
Age (year)	71.14 ± 8.00	72.00 ± 8.16	-1.84	0.066
Education (year)	8.77 ± 4.93	10.35 ± 4.96	-5.87	≤0.001^∗^
Smoking (yes/no)	385/146	387/551	132.76	≤0.001^∗^
Tea drinking (yes/no)	391/140	532/406	41.56	≤0.001^∗^
Hypertension (yes/no)	257/274	435/503	0.56	0.455
Hyperlipidemia (yes/no)	82/449	164/774	1.01	0.314
Diabetes (yes/no)	78/453	167/771	2.37	0.124
Traumatic brain injury (yes/no)	29/502	42/896	0.71	0.398
Baseline MoCA	21.56 ± 5.71	22.49 ± 5.70	3.21	0.073
MoCA naming	2.38 ± 0.84	2.54 ± 0.77	5.82	0.016^∗^
MoCA abstraction	0.86 ± 0.84	1.05 ± 0.83	4.70	0.030^∗^

*p* < 0.05 was considered statistically significant.

**Table 2:** Regression analysis of cognitive function.

Model	Baseline MoCA naming function		Baseline MoCA abstraction function		Follow-up MoCA naming function	
	*B*	*p* value	*B*	*p* value	*B*	*p* value
Age (year)	-0.01	0.000^∗^	-0.02	0.000^∗^	-0.02	≤0.001^∗^
Education (year)	0.06	0.000^∗^	0.07	0.000^∗^	0.06	≤0.001^∗^
Hypertension (yes/no)	0.01	0.912	0.11	0.010^∗^	0.05	0.327
Diabetes (yes/no)	-0.01	0.94	0.12	0.030^∗^	0.07	0.289
Traumatic brain injury (yes/no)	-0.09	0.338	-0.22	0.020^∗^	-0.03	0.794
Drinking (yes/no)	0.10	0.016^∗^	0.10	0.031^∗^	0.16	0.003^∗^

*p* < 0.05 was considered statistically significant.

**Table 3:** Demographic, lifestyle, physical illness, and cognitive function of community male elderly at follow-up.

	Drinking (*n* = 354)	Nondrinking (*n* = 666)	*F* or *χ*^2^	*p* value
Age (year)	70.87 ± 7.96	72.12 ± 8.03	-2.20	0.028^∗^
Education (year)	8.99 ± 4.84	10.41 ± 4.88	-4.44	≤0.001^∗^
Smoking (yes/no)	254/100	281/385	80.98	≤0.001^∗^
Tea drinking (yes/no)	265/89	379/287	32.00	≤0.001^∗^
Hypertension (yes/no)	172/182	314/352	0.19	0.661
Hyperlipidemia (yes/no)	49/305	124/542	3.75	0.053
Diabetes (yes/no)	46/308	120/546	4.28	0.039^∗^
Traumatic brain injury (yes/no)	13/341	31/635	0.54	0.462
Follow-up MoCA	22.09 ± 5.93	22.48 ± 6.19	0.65	0.421
MoCA score decline	0.24 ± 3.377	0.00 ± 3.891	0.474	0.491
Follow-up MoCA naming	2.33 ± 0.88	2.53 ± 0.76	5.57	0.018^∗^
MoCA naming score decline	0.00 ± 0.000	−0.00 ± 0.494	1.597	0.207
MoCA abstraction score decline	−0.14 ± 0.874	−0.11 ± 0.843	0.074	0.786
New onset rate of dementia (%)	3.1%	4.7%	1.40	0.236

*p* < 0.05 was considered statistically significant.

## Data Availability

The data supporting our findings can be requested by emailing the corresponding author.

## Additional Points

*Summary Box*. Light-to-moderate alcohol consumption had no significant impact on the overall cognitive function and dementia risk of elderly males.
